# Understanding Coke Deposition Vis-à-Vis DRM Activity over Magnesia-Alumina Supported Ni-Fe, Ni-Co, Ni-Ce, and Ni-Sr Catalysts

**DOI:** 10.3390/nano13212874

**Published:** 2023-10-30

**Authors:** Yousef M. Alanazi, Naitik Patel, Anis H. Fakeeha, Jehad Abu-Dahrieh, Ahmed A. Ibrahim, Ahmed E. Abasaeed, Rawesh Kumar, Ahmed Al-Fatesh

**Affiliations:** 1 Chemical Engineering Department, College of Engineering, King Saud University, P.O. Box 800, Riyadh 11421, Saudi Arabia; yalanazi1@ksu.edu.sa (Y.M.A.); anishf@ksu.edu.sa (A.H.F.); aididwthts2011@gmail.com (A.A.I.); abasaeed@ksu.edu.sa (A.E.A.); 2 Department of Chemistry, Indus University, Ahmedabad 382115, India; naitikvagdoda@gmail.com (N.P.); kr.rawesh@gmail.com (R.K.); 3 School of Chemistry and Chemical Engineering, Queen’s University Belfast, Belfast BT9 5AG, UK

**Keywords:** MgO-Al_2_O_3_ support, Ni-Ce, Ni-Co, Ni-Fe, Ni-Sr, coke deposit mechanism

## Abstract

The catalytic conversion of CH_4_ and CO_2_ into H_2_-rich syngas is known as the dry reforming of methane (DRM). The dissociation of CH_4_ over active sites, coupled with the oxidation or polymerization of CH_4−x_ (x = 1–4), plays a crucial role in determining in determining the DRM product yield and coke deposition. Herein, a series of bimetallic-supported catalysts are prepared by the dispersion of Ni-M (M = Ce, Co, Fe, and Sr) over 60 wt% MgO-40 wt% Al_2_O_3_ (60Mg40Al) support. Catalysts are tested for DRM and characterized with XRD, surface area and porosity, temperature-programmed reduction/desorption, UV−VIS−Raman spectroscopy, and thermogravimetry. 2.5Ni2.5Sr/60Mg40Al and 2.5Ni2.5Fe/60Mg40Al, and 2.5Ni2.5Ce/60Mg40Al and 2.5Ni2.5Co/60Mg40Al have similar CO_2_ interaction profiles. The 2.5Ni2.5Sr/60Mg40Al catalyst nurtures inert-type coke, whereas 2.5Ni2.5Fe/60Mg40Al accelerates the deposition of huge coke, which results in catalytic inferiority. The higher activity over 2.5Ni2.5Ce/60Mg40Al is due to the instant lattice oxygen-endowing capacity for oxidizing coke. Retaining a high DRM activity (54% H_2_-yield) up to 24 h even against a huge coke deposition (weight loss 46%) over 2.5Ni2.5Co/60Mg40Al is due to the timely diffusion of coke far from the active sites or the mounting of active sites over the carbon nanotube.

## 1. Introduction

The concentration of greenhouse gases has reached a critical level, and the effects are being seen everywhere. Apart from the rise in sea level, drastic seasonal changes, droughts, and wildfires are also becoming intensified across the globe. At this point, the catalytic conversion of the two leading greenhouse gases, CH_4_ and CO_2_, into H_2_-rich syngas is important. The proper catalyst for DRM may be a game changer for depleting greenhouse gases from the environment, and one of the main products, H_2_, is considered to be clean energy. This reaction is known as the dry reforming of methane (DRM), and it is highly endothermic (CH_4_ + CO_2_ → 2H_2_ + 2CO; ΔH° = 247.34 kJ/mol). Pt, Pd, Ir, Ru, and Ni dispersed over different supports such as MgO, CeO_2_, La_2_O_3_, Y_2_O_3_, TiO_2_, Si_2_O_3_, Al_2_O_3,_ and ZrO_2_ are largely investigated for this reaction [1]. 

The selection of Ni as the active site and MgO, Al_2_O_3,_ and TiO_2_ as the support is cost effective. Among the supports, TiO_2_ is known for its stronger metal−support interaction. Still, the catalytic performance of the Ni/TiO_2_ catalyst was found to be inferior to the Ni/MgO and Ni/Al_2_O_3_ catalysts due to the destabilization of titania phases, as well as the coverage of active sites of Ni by TiO_x_ species [2,3,4,5,6,7]. Ni/MgO forms a NiO-MgO solid solution, which becomes hard at higher temperatures and results in a lower reducibility of NiO. It turns the inferior catalysts into the Ni/Al_2_O_3_ catalyst [4,5,6,7]. The Ni/Al_2_O_3_ catalyst was found to be superior compared with all of the above catalysts. However, an enriched surface acid profile vis-à-vis massive coke deposition limits its optimum catalytic performance on an industrial scale [8]. On the other hand, the addition of MgO with Al_2_O_3_ has been found to inhibit the diffusion of Ni active sites into Al_2_O_3_ (by forming NiAl_2_O_4_), and stabilizes Ni particles over MgAl_2_O_4_. MgO also depletes the acidity of Al_2_O_3_ and resists carbon deposition [9]. 

How can the catalytic limitation over the Ni/Al_2_O_3_ catalyst or Ni/MgO be overcome? Can we decrease the acidity of the support in the Ni/Al_2_O_3_ catalyst by incorporating some amount of MgO? Can we retain the reducibility of NiO (above 700 °C) over MgO by adding Al_2_O_3_? Keeping the major framework of basic MgO and rest by Al_2_O_3_, both DRM’s favorable properties of inherent basicity and Ni stability can be retained above 700 °C DRM temperature. Such an approach was previously tested for DRM by Quan et al. by keeping a Mg/Al ratio of 1:3 [8]. So, the dispersion of Ni over the MgO-Al_2_O_3_ support may be a promising approach for DRM. Further, the catalytic performance can be elevated by using suitable promotors over the Ni/MgO-Al_2_O_3_ catalyst. 

Because of its inherent basic property vis-à-vis CO_2_ interactions, alkaline earth metals are frequently used as promoters over alumina-supported Ni catalysts. The Sr-promoted alumina-supported Ni catalyst showed enhanced basicity and a more significant metal support interaction by forming NiAl_2_O_4_ [10]. Alipour et al. found that Mg, Ba, and Ca-modified Ni/Al_2_O_3_ had a higher reducibility, coke resistance, and activity in the order of Mg > Ba > Ca (than Ni/Al_2_O_3_ catalyst) [11]. Karam et al. claimed the enhanced formation of small metallic Ni by spinel nickel-aluminate nano species by adding Mg over an alumina-supported Ni catalyst [12]. Diyanath et al. showed an enhanced metal−support interaction of Ni over magnesia-alumina support [13]. Akki et al. revealed that ceria-promoted MgAl_2_O_4_-supported Ni catalyst attained a highly reducible surface with enhanced basicity and effective active oxygen transfer [14]. Iron incorporation over an alumina-supported Ni catalyst was found to inhibit the deposition of inactive carbon and the oxidation of metallic Ni [15]. Previously, the promotional addition of Co over a magnesia-alumina -supported Ni catalyst resulted in enhanced coke elimination due to the high oxygen affinity of cobalt [16]. 

A further literature survey reveals the promotional role of Ce, Co, Fe, and Sr towards DRM. Herein, 2.5 wt% Ni-2.5 wt% M (M = Ce, Co, Fe, Sr) is supported over 60 wt% MgO-40 wt%Al_2_O_3_. The catalyst system is investigated for DRM and is characterized thoroughly via X-ray diffraction, surface area and porosity measurement, H_2_-temperature programmed reduction, CO_2_-temperature programmed desorption, UV−VIS spectroscopy, Raman spectroscopy, and thermogravimetry. Here, the good correlation of characterization results with the catalytic activity is aimed at bringing about more insight into two major competitive routes, DRM reaction and coke deposition, through four sets of bimetallic metal sites (Ni-Co, Ni-Fe, Ni-Sr, and Ni-Ce) over a cheap magnesia-alumina support. It may also pave the way for developing a robust DRM catalyst system using cheap chemicals like MgO, Al_2_O_3_ nickel nitrate, iron nitrate, strontium nitrate, and cobalt nitrate. 

## 2. Materials and Methods

### 2.1. Materials

Nickel nitrate hexahydrate, cobalt nitrate hexahydrate, iron nitrate nonahydrate, cerium nitrate hexahydrate, strontium nitrate hexahydrate, MgO, and Al_2_O_3_.

### 2.2. Catalyst Preparation 

Here, 0.95 g of 60 wt%MgO-40 wt%Al_2_O_3_ support was dispersed in 100 mL of deionized water at room temperature for 20 min. The nitrate precursor of Ni (equivalent to 2.5 wt%) and nitrate precursor of one metal (equivalent to 2.5 wt%) were added to the dispersed support solution at 80 °C until dry. The obtained solid sample was first calcined in air at 600 °C for 3 h. The support was abbreviated as 60Mg40Al, and the Ni supported catalysts were abbreviated as 2.5Ni-2.5M/60Mg40Al (M = Ce, Co, Fe, and Sr). 

### 2.3. Catalyst Characterization

Brunauer–Emmett–Teller (BET), X-ray diffraction (XRD), Raman spectroscopy, H_2_ temperature-programmed reduction (H_2_-TPR), CH_4_-temperature programmed surface reaction (CH4-TPSR), CO_2_ temperature-programmed desorption (CO_2_-TPD), transmission electron microscopy (TEM), and thermogravimetric analysis (TGA) were used to characterize the catalysts. The Supporting Information (S1) provides a thorough explanation of the instruments and the characterization process.

### 2.4. Catalyst Activity Test 

The dry reforming of the methane experiment was carried over 0.1g of promoted catalysts at 700 °C under 1atm pressure in a stainless steel vertical fixed tubular reactor (PID Eng. & Tech Micro Activity Reference, 9.1 mm i.d. and 30 cm long). The temperature of the reactor was monitored by an axially positioned thermocouple (K-type stainless sheathed) at the center of the catalyst bed. Before the catalytic tests, reductive pretreatment of the catalyst samples was carried out under the flow of hydrogen (20 mL/min) for 60 min at 600 °C. The mixture of gases fed CH_4_/CO_2_/N_2_ in the respective volumes as 6:6:2, and the volume flow rate was 70 mL·min^−1^ and 42,000 mL(h·g_cat_) ^−1^ gas hourly space velocity was passed through the reactor. The product gas stream was analyzed with a GC (GC-2014 Shimadzu) unit equipped with a thermal conductivity detector and two columns, Porapak Q and Molecular Sieve 5A. H_2_ yield % and H_2_/CO molar ratio are determined using the following expressions:

$$H_{2}\;\mathrm{yield}\;(\%)=\frac{Mole\;of\;H_{2}\;in\;Product}{2*Mol\;of\;{{CH}_{4}}_{in}}\times 100\%$$

$$\mathrm{CO}\;\mathrm{yield}\;(\%)=\frac{Mole\;of\;CO\;in\;Product}{Mol\;of\;{{CH}_{4}}_{in}\;+\;Mol\;of\;{{CO}_{2}}_{in}}\times 100\%$$

## 3. Results and Discussion

### 3.1. Characterization Result and Discussion

**Figure 1:**
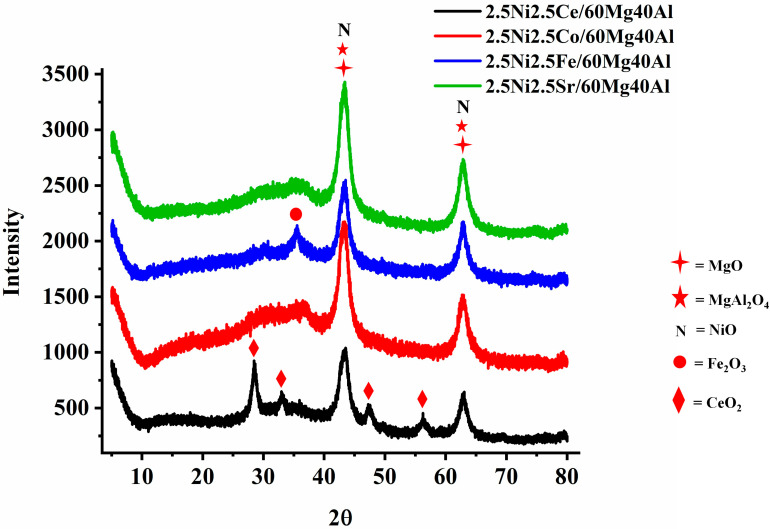
X-ray diffraction (XRD) of the 2.5Ni-2.5M/60Mg40Al (M = Ce, Co, Fe, and Sr) catalysts.

The X-ray diffraction patterns of 2.5Ni-2.5M/60Mg40Al (M = Ce, Co, Fe, Sr) are shown in Figure 1. 2.5 wt%Ni-2.5 wt%M/60Mg40Al (M = Ce, Co, Fe, Sr) retained a noticeable diffraction pattern for the cubic NiO phase (JCPDS reference number 01-073-1519), cubic MgO phase (JCPDS reference number 01-075-1525), and orthorhombic MgAl_2_O_4_ phase (JCPDS reference number 00-033-0853) about 43.16°, 62.4° Bragg’s angle. The 2.5 wt%Ni-2.5 wt%Ce/60Mg40Al catalyst has additional diffraction pattern for cubic CeO_2_ (at Bragg’s angle 2θ = 28.55°, 33.08°, 47.48°, 56.34°; JCPDS reference number 01-075-0390) and the 2.5 wt%Ni-2.5 wt%Fe/60Mg40Al catalyst had an additional diffraction pattern for cubic Fe_3_O_4_ (at Bragg’s angle 2θ = 30.08°, 35.43°; JCPDS reference number 01-075-1372). 

**Figure 2:**
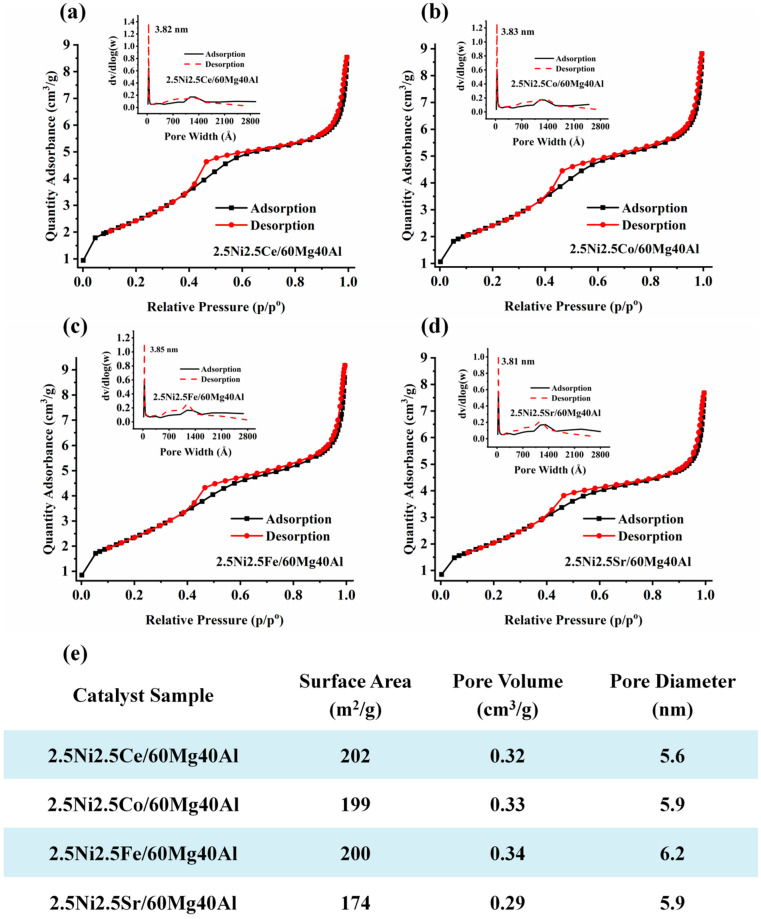
The surface area and porosity of (**a**) 2.5Ni2.5Ce/60Mg40Al, (**b**) 2.5Ni2.5Co/60Mg40Al, (**c**) 2.5Ni2.5Fe/60Mg40Al, and (**d**) 2.5Ni2.5Sr/60Mg40Al. (**e**) Table of surface area, pore volume, and pore diameter of the 2.5Ni-2.5M/60Mg40Al (M = Ce, Co, Fe, Sr) catalysts.

The surface and porosity results of the 2.5Ni-2.5M/60Mg40Al (M = Ce, Co, Fe, Sr) catalysts are shown in Figure 2. All catalysts showed type IV isotherms with an H4 hysteresis loop, indicating the presence of both micropores and mesopores [17]. The 2.5Ce2.5M/60Mg40Al (M = Ce, Co, Fe) catalyst had similar surface parameters (surface area 199–202 m^2^/g and pore volume 0.32–0.34 cm^3^/g). The surface area and pore volume of the 2.5Ni2.5Sr/60Mg40Al catalyst were the lowest (surface area: 174 m^2^/g, pore volume: 0.29 cm^3^/g) compared with the other catalysts. This indicates the serious accumulation of NiO and Sr compounds inside the pores. 

**Figure 3:**
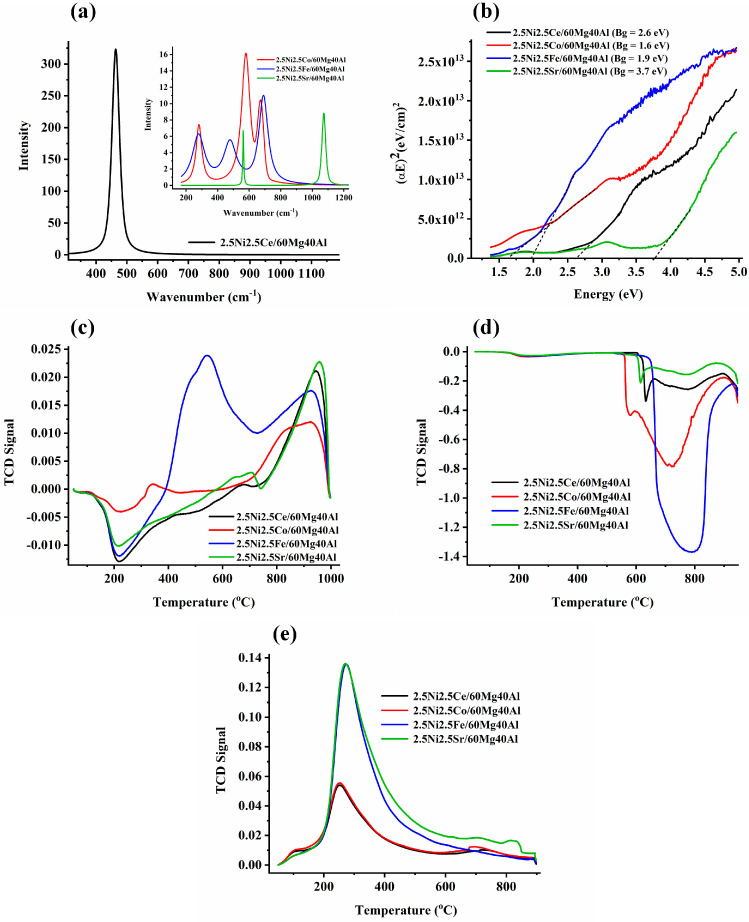
(**a**) The Raman spectra of the 2.5Ni-2.5M/60Mg40Al (M = Ce, Co, Fe, Sr) catalysts. (**b**) Bandgap of the 2.5Ni-2.5M/60Mg40Al (M = Ce, Co, Fe, Sr) catalysts. (**c**) H_2_-temperature programmed reduction profile of the 2.5Ni-2.5M/60Mg40Al (M = Ce, Co, Fe, Sr) catalysts. (**d**) CH_4_-temperature programmed surface reaction experiment of the 2.5Ni-2.5M/60Mg40Al (M = Ce, Co, Fe, Sr) catalysts. (**e**) CO_2_-temperature programmed desorption of the 2.5Ni-2.5M/60Mg40Al (M = Ce, Co, Fe, Sr) catalysts.

The Raman spectra of the 2.5Ni2.5M/60Mg40Al (M = Ce, Co, Fe, Sr) catalysts are shown in Figure 3.

The 2.5Ni2.5Ce/60Mg40Al catalyst showed an intense RAMAN band about 463 cm^−1^, which is attributed to the symmetric F_2g_ stretching mode of oxygen surrounding the Ce^4+^ in cubic CeO_2_ [18,19] (Figure 3a). The XRD of 2.5Ni2.5Ce/60Mg40Al also showed the prominent peak for cubic CeO_2_. The 2.5Ni2.5Sr/60Mg40Al catalyst showed two sharp Raman peaks at 560 cm^−1^ and 1071 cm^−1^ for the Sr-O and CO_3_^2−^ vibration of Sr^2+^, respectively [20]. The absence of Sr-related phases in XRD may be due to the good dispersion of the Sr compound over the catalyst surface. The 2.5Ni2.5Co/60Mg40Al catalyst showed Raman vibration bands at 282 cm^−1^, 580 cm^−1^, and 672 cm^−1^. In the literature, the Raman peaks for Co(OH)_2_ have been reported at 284 cm^−1^, 462 cm^−1^ (OCoO bending mode), and 522 cm^−1^ (OH deformation mode) [21,22]. Previously, in the Ni_1-x_Co_x_TiO_3_ sample, as per the varying degree of x from 0.05 to 0.80, the Raman band was found to shift from 289 to 272 cm^−1^ [23]. This means for the 2.5Ni2.5Co/60Mg40Al catalyst, the Raman peak of about 284 cm^−1^ was for the Co(OH)_2_ vibration and was very sensitive to the degree of substitution. The Raman peak at 580 cm^−1^ was reported for the vibration mode of CoO(OH) (oxidation state of Co (III)) [21]. The peak at 580 cm^−1^ was the most intense Raman peak in the 2.5Ni2.5Co/60Mg40Al catalyst. The Raman band at 675–680 cm^−1^ was a typical band reported for Co-O vibration in CoO, Co_3_O_4_, and CoO·xCoO [22,24,25,26]. From the Raman analysis, an interacted-CoO, Co_3_O_4_, Co (OH)_2,_ and CoO (OH)-type composition can be claimed. Previously, the absence of the CoO (OH) pattern in XRD was due to its amorphous nature [21]. It is noticeable that, here, the catalyst was prepared at 600 °C, activated at 800 °C, and employed for the DRM reaction at 700 °C. During this temperature range, various types of interconversion between cobalt compounds may be possible [22], as per the following reactions: 6Co(OH)_2_ + O_2_ → 2Co_3_O_4_ + 6H_2_O (at 120 °C), 12CoO(OH) → 4Co_3_O_4_ + O_2_ + 6H_2_O (at 252 °C), 2Co_3_O_4_ → 6CoO + O_2_ (at 790 °C). Here, H_2_-TPR was taken after the catalyst preparation. This means that over a fresh 2.5Ni2.5Co/60Mg40Al catalyst, some complex structure of cobalt compound made up of 6Co (OH)_2_ and Co_3_O_4_ (or Co_2_O_3_·CoO) may be present. In XRD, the phase for NiO was observed, but no phases of the cobalt compound were found. So, it can be expected that the cobalt compound may be amorphous or the cobalt compound may be more dispersed than NiO over the catalyst. The 2.5Ni2.5Fe/60Mg40Al catalyst had Raman bands at 280 cm^−1^, 480 cm^−1^, and 689 cm^−1^. In the literature, peaks at 290 cm^−1^, 490 cm^−1^, and 659 cm^−1^ were reported for the Raman vibration of Fe_2_O_3_ [27]. The red and blue shift of the Raman band (concerning the standard Fe_2_O_3_ band) was due to the insertion of the hetero-atom in the lattice of Fe_2_O_3_. The band at 689 cm^−1^ may be related to structural disorder due to the insertion of the Al cation into the iron oxide lattice [28].

The bandgap of the UV−VIS results is shown in Figure 3b. Among all of the catalysts, the band gap between the valance band and conduction band was found to be lowest for the 2.5Ni2.5Co/60Mg40Al catalyst and highest for 2.5Ni2.5Sr/60Mg40Al. The H_2_-temperature programmed reduction profile (H_2_-TPR) of 2.5Ni2.5M/60Mg40Al (M = Ce, Co, Fe, Sr) catalysts are shown in Figure 3c. The negative H_2_-TPR in the temperature range of 200 °C was attributed to hydrogen spillover into the mesopores [29]. Upon the incorporation of Ce or Sr along with Ni over 60Mg/40Al, there was a single intense peak of about 900 °C, which was attributed to reducible “strongly interacted NiO” species [30]. The H_2_-TPR profile of the 2.5Ni2.5Fe/60Mg40Al catalyst was unique. It had merge peak at 500 °C and 550 °C and a peak at 850 °C. These peaks were attributed to the reduction in “moderately interacted NiO” species (into Ni), reduction in Fe_3_O_4_ (into FeO), and “strongly interacted NiO” species (into Ni), respectively [30]. It may be possible that the reduction peak of FeO to Fe may be merged with a peak of about 850 °C over the 2.5Ni2.5Fe/60Mg40Al catalyst. So, the peak intensity at 850 °C may be due to reducible strongly-interacted-NiO and reducible FeO species. 

The CH_4_-temperature programmed surface reaction (TPSR) experiment was carried out up to a temperature of 950 °C for the different catalyst systems (Figure 3d). The CH_4_-TPSR peaks at different temperatures signified the extent of CH_4_ decomposition over different active sites at the catalyst. From the CH_4_-TPSR experiments, it was clear that 2.5Ni2.5Fe/60Mg40Al had the highest density for the CH_4_ decomposition sites, whereas 2.5Ni2.5Ce/60Mg40Al and 2.5Ni2.5Sr/60Mg40Al had the least sites for CH_4_ decomposition [31]. The CO_2_-temperature programmed desorption profile of the 2.5Ni2.5M/60Mg40Al (M = Ce, Co, Fe, Sr) catalysts is shown in Figure 3e. All samples showed a desorption peak at about 300 °C, attributed to surface basicity due to surface oxygen anion [32]. The desorption peak intensity for the 2.5Ni2.5Fe/60Mg40Al and 2.5Ni2.5Sr/60Mg40Al catalysts indicated a similar type of basic site density over both catalysts. In the same way, the basic site distribution over the 2.5Ni2.5Ce/60Mg40Al and 2.5Ni2.5Co/60Mg40Al catalysts was similar. The total basic sites over the 2.5Ni2.5M/60Mg40Al (M = Sr, Fe) catalyst was higher than the 2.5Ni2.5M/60Mg40Al (M = Co, Ce) catalyst. Overall, it can be said that the CO_2_ interaction of the 2.5Ni2.5Fe/60Mg40Al and 2.5Ni2.5Sr/60Mg40Al catalysts was more significant than the 2.5Ni2.5Ce/60Mg40Al and 2.5Ni2.5Ce/60Mg40Al catalysts. 

**Figure 4:**
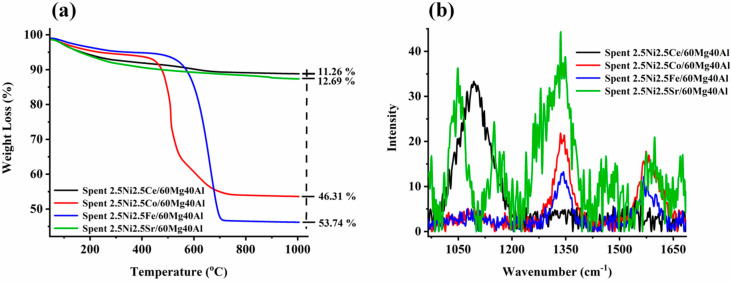
(**a**) Thermogravimetry profile of spent-2.5Ni-2.5M/60Mg40Al (M = Ce, Co, Fe, and Sr) catalysts. (**b**) Raman spectra of spent-2.5Ni-2.5M/60Mg40Al (M = Ce, Co, Fe, and Sr) catalysts.

The thermogravimetry profile of spent-2.5Ni2.5M/60Mg40Al (M = Ce, Co, Fe, Sr) is shown in Figure 4a. The minimum weight loss (~11%) was noticed for the spent-2.5Ni2.5Ce/60Mg40Al catalyst. It is noticeable that the CO_2_-desorption profiles of both the 2.5Ni2.5Co/60Mg40Al catalyst and 2.5Ni2.5Ce/60Mg40Al catalyst were similar, but the amount of oxidizable carbon deposit earlier was much higher than later. This indicates the different oxidizing capacities of both catalysts, even though both had the same level of interaction with CO_2_ (verified by CO_2_-TPD). This indicates a different oxidizing route than the direct oxidation by CO_2_. The instant oxygen-endowing capacity of the ceria-based catalyst system ignited carbon deposits before CO_2_. So, the delay in carbon deposit oxidation by CO_2_ was minimized, and the catalyst surface had less carbon deposit than the 2.5Ni2.5Ce/60Mg40Al catalyst. The low weight loss (12.69%) over the spent-2.5Ni2.5Sr/60Mg40Al catalyst may be related to the presence of inert carbon species/non-oxidizable carbon species. The spent-2.5Ni2.5Fe/60Mg40Al and spent-2.5Ni2.5Co/60Mg40Al catalyst had a serious weight loss of 53.74% and 46.31%, respectively. In H_2_-TPR, we found huge reduction peaks for iron, and the oxidation of lower iron states was not neglected during TGA of spent-2.5Ni2.5Fe/60Mg40Al catalyst. Even with the possibility of oxidation of Fe, Spent-2.5Ni2.5Fe/60Mg40Al had the highest weight loss at 53.75%. This means the actual weight loss due to carbon deposit over Spent-2.5Ni2.5Fe/60Mg40Al was even higher. To understand the type of carbon deposit, the Raman profile of spent-2.5Ni2.5M/60Mg40Al (M = Ce, Co, Fe, Sr) catalysts was also carried out and is shown in Figure 4b and Figure S1. The peak profile at 1340 cm^−1^ was attributed to an imperfect carbon band/disordered carbon band (I_D_), whereas the peak at 1570 cm^−1^ was signified by the ordered graphitic carbon band (I_G_) [33]. The D and G bands were due to sp^2^ carbon vibration. Interestingly, ceria-based catalyst spent-2.5Ni2.5Ce/60Mg40Al had no band for graphitic/ordered carbon or disordered carbon, but it had a broad peak at 1070 cm^−1^ due to C-C sp^3^ vibration (T band) [33]. Including overtone bands, the spent-2.5Ni2.5Ce/60Mg40Al catalyst also had a 2D′′ band (2435 cm^−1^) [33] and the 2D band (2676 cm^−1^) [33]. The 2.5Ni2.5Fe/60Mg40Al and 2.5Ni2.5Co/60Mg40Al catalysts had only D, G, and 2D bands. The intensity of the disordered carbon band increased in the following order: spent-2.5Ni2.5Fe/60Mg40Al < spent-2.5Ni2.5Ce/60Mg40Al < spent-2.5Ni2.5Co/60Mg40Al < spent-2.5Ni2.5Sr/60Mg40Al. In the same way, the intensity of the graphitic carbon band increased in the following order: spent-2.5Ni2.5Fe/60Mg40Al < spent-2.5Ni2.5Ce/60Mg40Al < spent-2.5Ni2.5Co/60Mg40Al ~ spent-2.5Ni2.5Sr/60Mg40Al. The spent-2.5Ni2.5Sr/60Mg40Al catalyst had many additional bands, such as the T band (split into 1050 cm^−1^ and 1080 cm^−1^), 2T band (2100 cm^−1^), carbyne band (2175 cm^−1^) [34] 2D′′ band (2435 cm^−1^), and G + D band (at 2900 cm^−1^) [33]. Overall, it seems that the 2.5Ni2.5Sr/60Mg40Al catalyst had a wide range of inert carbon species over the surface, which did not oxidize during TGA (giving low weight loss), but could seriously affect the catalytic activity.

**Figure 5:**
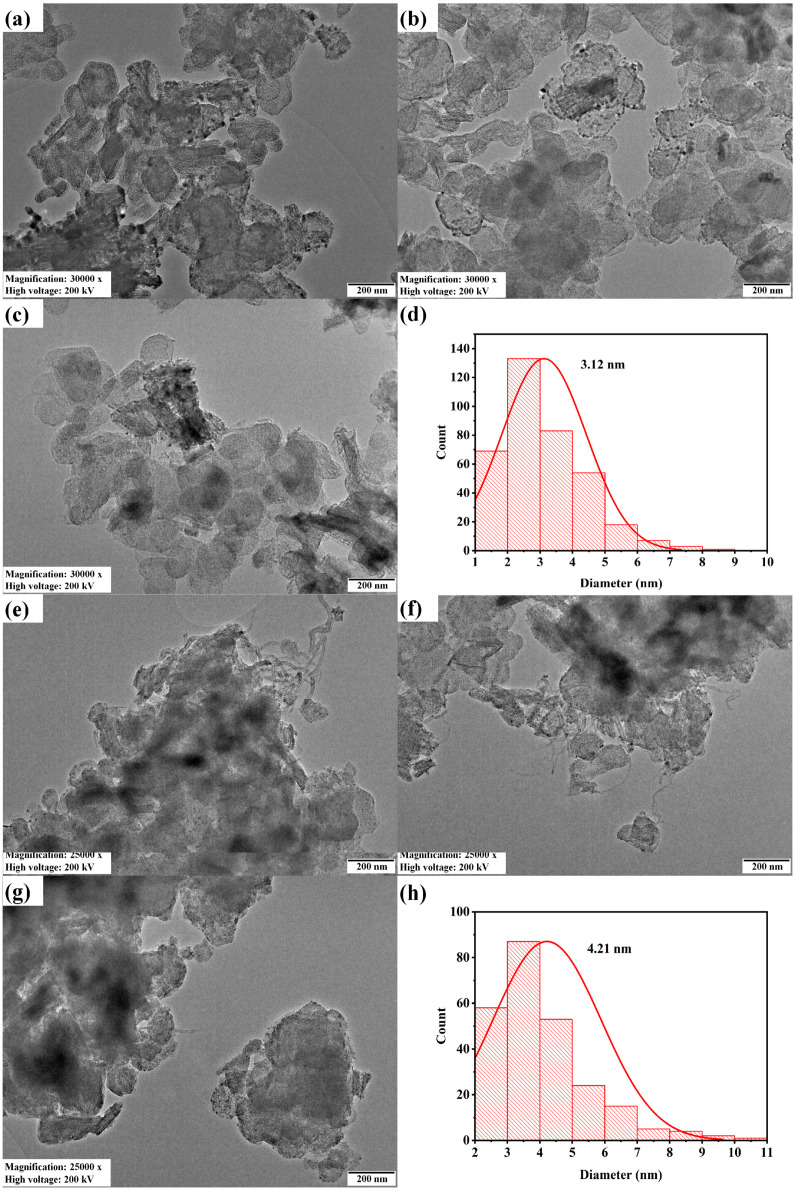
Transmission electron microscopy of the (**a**–**c**) 2.5Ni2.5Co/60Mg40Al catalyst at 30,000× magnification, 200 kV voltage and 200 nm scale; (**e**–**g**) spent-2.5Ni2.5Co/60Mg40Al catalyst at 25,000× magnification, 200 kV voltage and 200 nm scale; Ni particle size distribution; (**d**) 2.5Ni2.5Co/60Mg40Al catalyst (**h**). Spent-2.5Ni2.5Co/60Mg40Al catalyst.

The transmission electron microscopy and Ni particle size distribution of fresh and spent 2.5Ni-2.5Co/60Mg40Al catalysts are depicted in Figure 5. The particle size of the spent 2.5Ni-2.5Co/60Mg40Al catalyst was larger (4.21 nm) than the fresh 2.5Ni-2.5Co/60Mg40Al catalyst (3.12 nm). The dark shadow marks and carbon nanotubes were evident over the spent-2.5Ni-2.5Co/60Mg40Al catalyst. 

### 3.2. Catalytic Activity Result and Discussion

**Figure 6:**
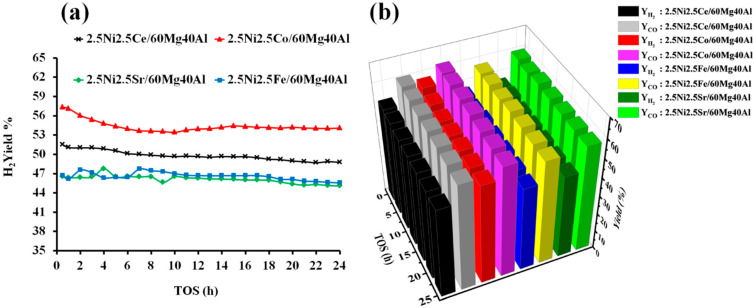
Catalytic activity results of 2.5Ni-2.5M/60Mg40Al (M = Ce, Co, Fe, and Sr) catalysts (**a**) H_2_-yield vs. TOS. (**b**) Bar diagram of H_2_ yield and CO yield vs. TOS.

The catalytic activity of the pure NiO and support-60Mg40Al towards dry reforming of methane were tested. The H_2_ yield and CO yield with pure NiO were found to be only 0.75% and 1.25%, respectively, at a reaction temperature of 700 °C. The support “60 wt%MgO40 wt%Al_2_O_3_” was also found to exhibit a catalytic activity, but it was much more selective towards CO than H_2_. 60Mg40Al showed 0.06% H_2_ yield and 4.10 CO yield. However, when Ni was dispersed over the 60Mg40Al support, the H_2_ yield eventually increased more than 60%. This suggests that the size and stability of Ni against high temperatures over proper support is essential for achieving a high DRM activity. The size and morphology of Ni can be controlled by proper calcination methodology and mixing a suitable matrix with Ni during the catalyst preparation [35,36]. The XRD results show that the Al_2_O_3_ and MgO metal oxide matrixes mixed, and the support obtained a new phase (orthorhombic MgAl_2_O_4_) identity that was composed of Mg, O, and Al. That means a homogenous matrix of Mg, O, and Al was ready to carry the NiO-MO_x_ (M = Ce, Co, Fe, Sr) metal oxide to catalyze the dry reforming of methane. The catalytic active sites of “metallic Ni” were derived from the reduction in “strongly interacted NiO” species over the 2.5Ni-2.5M/60Mg40Al (M = Ce, Co, Fe, Sr) catalyst system. The catalytic activity results of 2.5Ni-2.5M/60Mg40Al (M = Ce, Co, Fe, Sr) catalysts are shown in Figure 6. Among all of the catalysts, 2.5Ni2.5Co/60Mg40Al had a 57% H_2_ yield, which decreased to 54% after 24 h. The CO yield was above the H_2_ yield, and it remains between 62–60% 24 h TOS. The relatively higher CO yield than H_2_ yield indicates the presence of H_2_ consuming reactions. Over the catalytic surface, H_2_ can interact with carbon monoxide (CO) or carbon dioxide (CO_2_) and form water (H_2_O) and carbon (H_2_ + CO → H_2_O + C; 2H_2_ + CO_2_ → 2H_2_O + C). However, with a positive value for Gibb’s free energy, these reactions were not thermodynamic feasible [37]. On the other hand, the reaction of H_2_ and CO_2_ forming CO and water is thermodynamically feasible and is known as reverse water gas shift reaction. This reaction increases the concentration of CO at the expense of the H_2_ concentration, resulting into a higher CO yield. It is worth noting that we did not detect H_2_O in the gas-chromatogram, indicating the involvement of water in some other reactions. Thermodynamic equilibrium studies of DRM via Gibbs free energy have shown that the gasification of coke by water is a thermodynamically favorable reaction (C + H_2_O → CO + H_2_). Overall, the reverse water gas shift reaction and gasification reaction were also present along with the DRM reaction, resulting in an increase in CO concentration or CO yield [37]. The 2.5Ni2.5Ce/60Mg40Al catalyst showed a moderate activity towards DRM and H_2_ yield, and the CO yield remained between 51–49% and 60–59% during 24 h TOS, respectively. The activity of the 2.5Ni2.5Fe/60Mg40Al and 2.5Ni2.5Sr/60Mg40Al catalysts was found to be inferior, and their catalytic H_2_ yield remained about 45% during 24 h TOS. 

**Figure 7:**
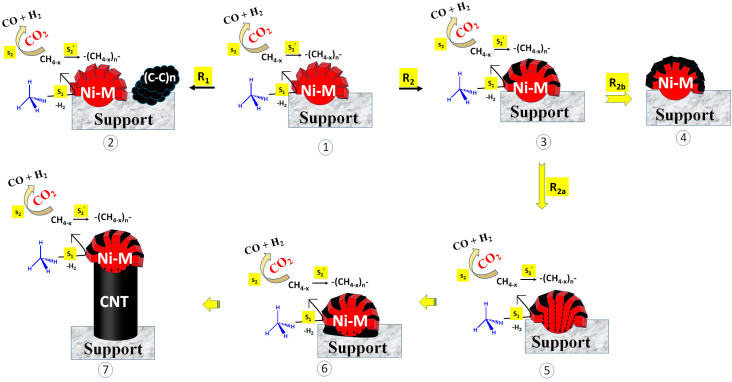
Different routes of coke deposition. Route 1 (R_1_): Decomposition of CH_4_ at catalytic active sites (into CH_4−x_), polymerization of CH_4−x_ (into coke), and diffusion of coke far from the active sites. Route 2 (R_2_): decomposition of CH_4_ at catalytic active sites (into CH_4−x_), polymerization of CH_4−x_ (into coke), and coke deposition into the cell gap of active sites (as Ni-metal). Sub-route R_2a_: Coke deposition inside Ni-M, precipitation at metal−support junction, and mounting of Ni over coke. Sub-route R_2b_: Coke deposition inside Ni-M, encapsulation of active sites (Ni-M). M = Fe, Co, Ce, Ni; S_1_: CH_4_ decomposition into CH_4−x_, S_2_: Oxidation of carbon deposit, S_2_′. polymerization of carbon deposit into coke.

Here, we discuss the catalytic routes and coke deposition routes over different catalyst surfaces. The possible coke deposition routes are shown in Figure 7. The catalyst may bear the sites for CH_4_ decomposition or CO_2_ decomposition [38,39,40]. Further, the decomposed species (reaction intermediate) were quite active and could interact with another species or the same species instantly. In the current catalyst system, metallic Ni sites were available for CH_4_ decomposition. It is well accepted that CH_4_ is decomposed into CH_4−x_ and H_2_ over metallic Ni (Step S1; Figure 7). After that, CH_4−x_ may be oxidized by CO_2_ (under DRM) into syngas (CO + H_2_) (Step S2, Figure 7). However, the delay in the oxidation of CH_4−x_ allowed for the polymerization of CH_4−x_ into coke (Step S2′; Figure 7). In a recent study, Zhang et al. carried out DFT studies focused on methane cracking over a catalyst surface and the subsequent growth of cracked carbon from the C1 carbon unit to the C6 carbon unit (C1–C6) [41]. From the C1–C4 carbon growth, “chain to chain” growth path (lengthening of carbon chain) was the main growth path. Further, at >C4, the growth path shifted from “chain to chain” to “chain to ring”. The growth energy of the “chain to ring” path was lower (1.5 eV) than the “chain to chain” path from C4–C5, and it was further lowered to 0.55 eV from C5–C6. Clearly, the C6 unit was formed majorly from chain to ring growth pathways. It is basic unit of complex structural carbon species such as graphene and carbon nanotube. 

The 2.5Ni2.5Co/60Mg40Al catalyst has massive coke deposition (weight loss 46%); these cokes are sp^2^ hybridized defective carbon (disordered type) and ordered carbon (graphitic types). However, the TEM image of the spent-2.5Ni2.5Co/60Mg40Al catalyst shows very few carbon nanotubes, but the impression of dark marks is frequently visible. The catalyst has the highest and constant catalytic activity (54% H_2_ yield from 14 h to 24 h reaction time) among the rest of the catalysts. The presence of few carbon nanotubes, the impression of frequent dark marks, massive weight loss, and the high catalytic activity of the 2.5Ni2.5Co/60Mg40Al catalyst indicate that carbon deposits are mostly accumulated as dark impressions over the catalyst, without affecting the catalytic active sites. If the rate of coke formation (at active sites) is firmly matched to the rate of coke diffusion (far from catalytic active sites) (Route 1; R_1_; Figure 7), then active sites remain exposed and coke is accumulated far from the active sites [42]. This means that activity is not affected, even with a high coke deposition. 

If the precursor of the carbon nanotube/C6 unit is not diffused out in a timely manner (far from the active site), it rapidly settles into the cell gap inside the Ni particle (or cell gap of active sites) (Route 2; R_2_; Figure 7). From here, there are two sub-routes. The first is the diffusion of coke inside the Ni particle, followed by the precipitation of coke at the site near the support. Slowly, the precipitated cokes are accumulated, the carbon nanotube grows, and, finally, catalytic active Ni is uplifted (R_2a_). In this way, metallic Ni is always mounted above the nanotube and remains exposed to the reactant. So, the activity of the catalyst is not suppressed. The second sub-route is coke deposition mounting in the cell gap and slowly encapsulating the active sites (metallic Ni) (R_2b_). Encapsulation of the active sites cuts the contact between the reactant and the catalyst. If step S2 (CH_4−x_ oxidation) is delayed over step S2′ (CH_4−x_ polymerization), encapsulation of the active sites is faster, and the catalytic activity is retarded. The 2.5Ni2.5Fe/60Mg40Al and 2.5Ni2.5Sr/60Mg40Al catalysts have a comparable activity (45–46% H_2_-yield) but inferior to the 2.5Ni2.5Ce/60Mg40Al and 2.5Ni2.5Co/60Mg40Al catalysts. The 2.5Ni2.5Fe/60Mg40Al catalyst has the same CO_2_ interaction profile as the 2.5Ni2.5Sr/60Mg40Al catalyst, but the bandgap energy (for electron transfer from the valance band to the conduction band) of 2.5Ni2.5Fe/60Mg40Al is about half that of 2.5Ni2.5Sr/60Mg40Al. The 2.5Ni2.5Fe/60Mg40Al catalyst is additionally populated with reducible iron oxide, which is known to trigger CH_4_ decomposition [42], but it has a huge coke deposition (~54% weight loss). The CH_4_-TPSR experiment also verifies the presence of the highest density of CH_4_ decomposition sites being in the 2.5Ni2.5Fe/60Mg40Al catalyst. The low catalytic activity (45% H_2_-yield) of 2.5Ni2.5Fe/60Mg40Al indicates that many of the active sites are arrested/encapsulated by bulk coke. The 2.5Ni2.5Sr/60Mg40Al catalyst has less surface area (and pore volume), which carries a diverse array of carbon deposits from the active to inert range (verified by Raman of spent catalyst). The shading of catalytic active sites by inert carbon over 2.5Ni2.5Sr/60Mg40Al causes the least catalytic activity (~45% H_2_-yield). The activity of the 2.5Ni2.5Ce/60Mg40Al catalyst is more progressed than the 2.5Ni2.5Fe/60Mg40Al and 2.5Ni2.5Sr/60Mg40Al catalysts due to its capability to release lattice oxygen instantly (prior than CO_2_) to oxidize carbon deposits. This results in the lowest non-graphitic carbon deposit and 49% H_2_ yield during 24 h TOS. 

## 4. Conclusions

The formation of mixed oxide phases (orthorhombic MgAl_2_O_4_ phase) by MgO-Al_2_O_3_ indicates a more homogeneous distribution of atoms (Mg, O, and Al) in the support material. The support serves as a carrier for NiO-MO_x_ (M = Ce, Co, Fe, and Sr) metal oxides, which catalyze the DRM reaction. The CO_2_ interaction profiles of both the 2.5Ni2.5Sr/60Mg40Al and 2.5Ni2.5Fe/60Mg40Al catalysts are similar, but the latter has less bandgap energy for electron transfer. Th relativelylow surface area and shading of active sites by inert carbon species make the 2.5Ni2.5Sr/60Mg40Al catalyst inferior (45% H_2_-yield). The presence of surface-active reducible iron oxide over 2.5Ni2.5Fe/60Mg40Al catalyst triggers pronounced CH_4_ decomposition, resulting in a huge carbon (inert and active) deposit (54% weight loss), which results in an inferior catalytic activity (47% H_2_-yield). Again, the CO_2_ interaction profiles of the 2.5Ni2.5Ce/60Mg40Al and 2.5Ni2.5Co/60Mg40Al catalysts are similar. The sudden interaction of lattice oxygen to the 2.5Ni2.5Ce/60Mg40Al catalyst causes the potential oxidation of coke. During 24 h on stream, the 2.5Ni2.5Ce/60Mg40Al catalyst achieves 49% H_2_ yield with minimal deposit of non-graphitic carbon. The 2.5Ni2.5Co/60Mg40Al catalyst system is characterized by the lowest bandgap for electron transfer and non-shading of the catalytic active sites, even with high coke deposition (weight loss of 46%). This is accomplished through the diffusion of coke far away from the active sites or by mounting the active sites over the carbon nanotubes. These surface modifications favor DRM, and the 2.5Ni2.5Ce/60Mg40Al catalyst shows a 54% H_2_ yield for up to 24 h in the stream test.

## Data Availability

Data is contained within the article.

## Supplementary Materials

The following supporting information can be downloaded at: https://www.mdpi.com/article/10.3390/nano13212874/s1, S1. The details of catalyst characterization, Figure S1: The Raman spectra of the spent-2.5Ni2.5M/60Mg40Al (M = Ce, Co, Fe, and Sr) catalyst.
