# Supplementary material for: Understanding Coke Deposition Vis-à-Vis DRM Activity over Magnesia-Alumina Supported Ni-Fe, Ni-Co, Ni-Ce, and Ni-Sr Catalysts
Source: Nanomaterials (Basel). 2023 Oct 30;13(21):2874. doi: 10.3390/nano13212874 (PMC10650252; doi:10.3390/nano13212874)
Supplement: Supplementary file 1 [file nanomaterials-13-02874-s001.zip › nanomaterials-2673236-supplementary.pdf]

Supplementary Materials

Understanding Coke Deposition Vis-à-Vis DRM Activity over Magnesia-Alumina Supported Ni-Fe, Ni-Co, Ni-Ce, and Ni-Sr Catalysts

S1. The details of catalyst characterization

X-ray diffraction (XRD) analysis was carried out in a Rigaku (Miniflex) diffractometer using Cu Ka radiation. The apparatus operated at 40 kV and 40 mA. The 2q scan range of 10-90 ° was used at a step of 0.02°. X'pert HighScore Plus software was used to examine the data file of the instrument. Different existing phases were matched with the ICDD (International Centre for Diffraction Data) data bank.

The Brunauer−Emmett−Teller (BET) surface area measurement of the catalysts and the support was performed in a Micromeritics Tristar II 3020 porosity and surface area analyzer, using N_2 isotherm (adsorption and desorption) data obtained at 77K. Before the analysis, the samples were degassed at 200°C for 3h to remove the adsorbed moisture and volatile gases. The perovskite catalysts' surface area and the pore size distribution were measured by N_2 adsorption–desorption at −196°C using a Micromeritics Tristar II 3020 for porosity and surface area analyzer.

At the end of the reaction, thermo gravimetric analysis (TGA) was carried out for the quantitative analysis of coke deposited on the catalysts used. The analysis was conducted with a thermo gravimetric/differential analyzer (Shimadzu TGA). About 0.015g of the sample was heated from room temperature up to 1000°C at a ramp of 20°C /min and the weight losses were recorded as the temperature increased.

A Laser Raman (NMR-4500) Spectrometer (JASCO, Japan) was used to obtain the Raman spectra of the spent catalyst samples. The wavelength of the excitation beam was set to 532 nm and an objective lens with 100x magnification was used for the measurement. The laser intensity was adjusted to 1.6 mW for 10s exposure time at three accumulations. This was to protect the sample from being damaged by laser irradiation. Measurement was performed in the range of 1200-3000 cm^{-1} (Raman shift) and the spectra were processed using Spectra Manager Ver.2 software (JASCO, Japan).

TPD automatic chemisorption equipment (Micromeritics Auto Chem II 2920, USA) was used to study the temperature-programmed reduction (TPR) and temperature-programmed desorption (TPD) of the catalysts. Around 70 mg of the sample was subjected to heat treatment for TPR at 10°C/min up to 900°C under atmospheric pressure and gas flow (30 mL/min) of 10% H_2/Ar mixture gas. For the TPD measurement, 70 mg of sample was used, which was first kept at 500°C for 1 h under helium flow to remove physically adsorbed species from the surface of the catalysts. After that, CO_2 adsorption was accomplished at 50°C for 30 min by passing 10% CO_2/He mixture gas at a flow rate of 30 mL/min. Moreover, the CO_2 desorption signal was subsequently recorded using TCD with a linear increase in temperature up to 800°C with a temperature ramp rate of 10°C/min.

For the H_2-TPR analysis, tests were conducted over a temperature range of 50-800°C and 2.40 L/h flow of 10% H_2/Ar mixture. During the H_2-TPR analysis of the catalyst, 0.070 g of the catalyst precursors were first heated to 150°C and held at that temperature for 60 min in the presence of Ar at a rate of 1.8 L/h and then

cooled to room temperature. Next, the sample temperature was raised to 900°C at 10 K/min under a 10% H_2/Ar mixture in an automatic furnace at 1 atm.

The catalyst morphology is measured using a 120 kV JEOL JEM-2100F (Akishima, Japan) transmission electron microscope (TEM).

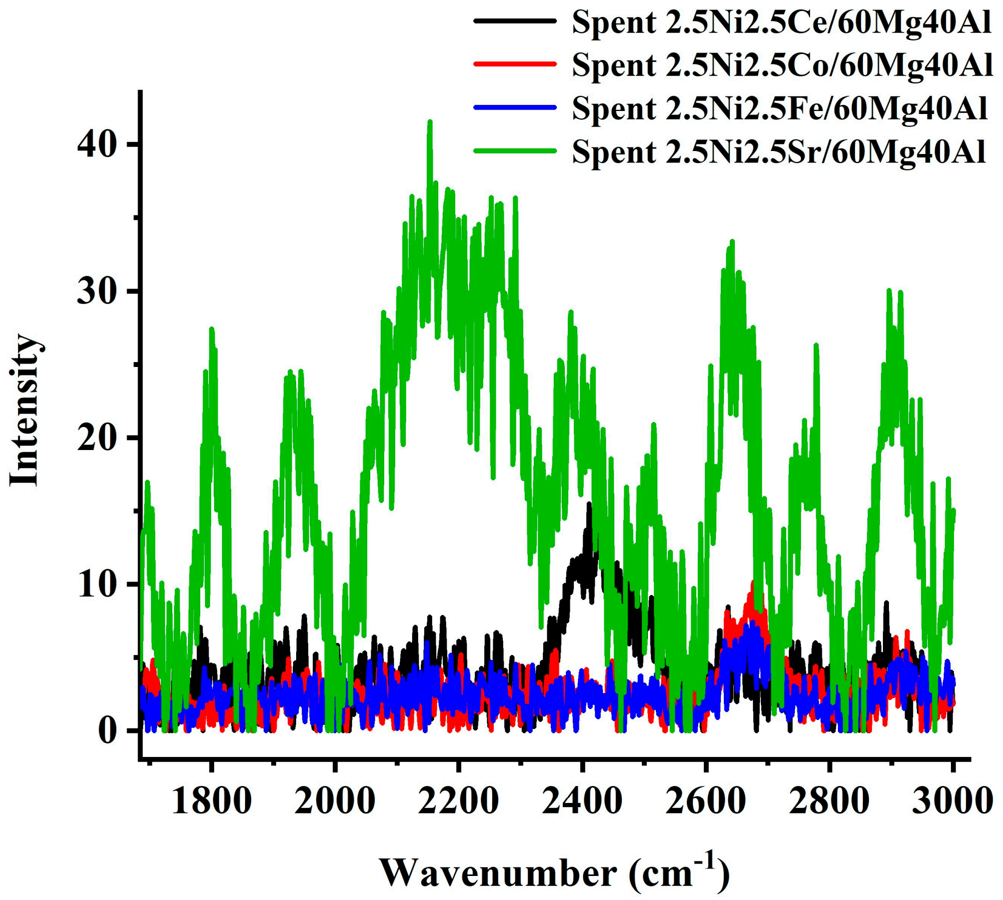

Figure S1. The Raman spectra of spent-2.5Ni2.5M/60Mg40Al (M = Ce, Co, Fe, Sr) catalyst
